# Supplementary material for: Studies on the Properties of the Sporulation Specific Protein Dit1 and Its Product Formyl Tyrosine
Source: J Fungi (Basel). 2020 Jun 3;6(2):77. doi: 10.3390/jof6020077 (PMC7345447; doi:10.3390/jof6020077)
Supplement: Supplementary file 1 [file jof-06-00077-s001.pdf]

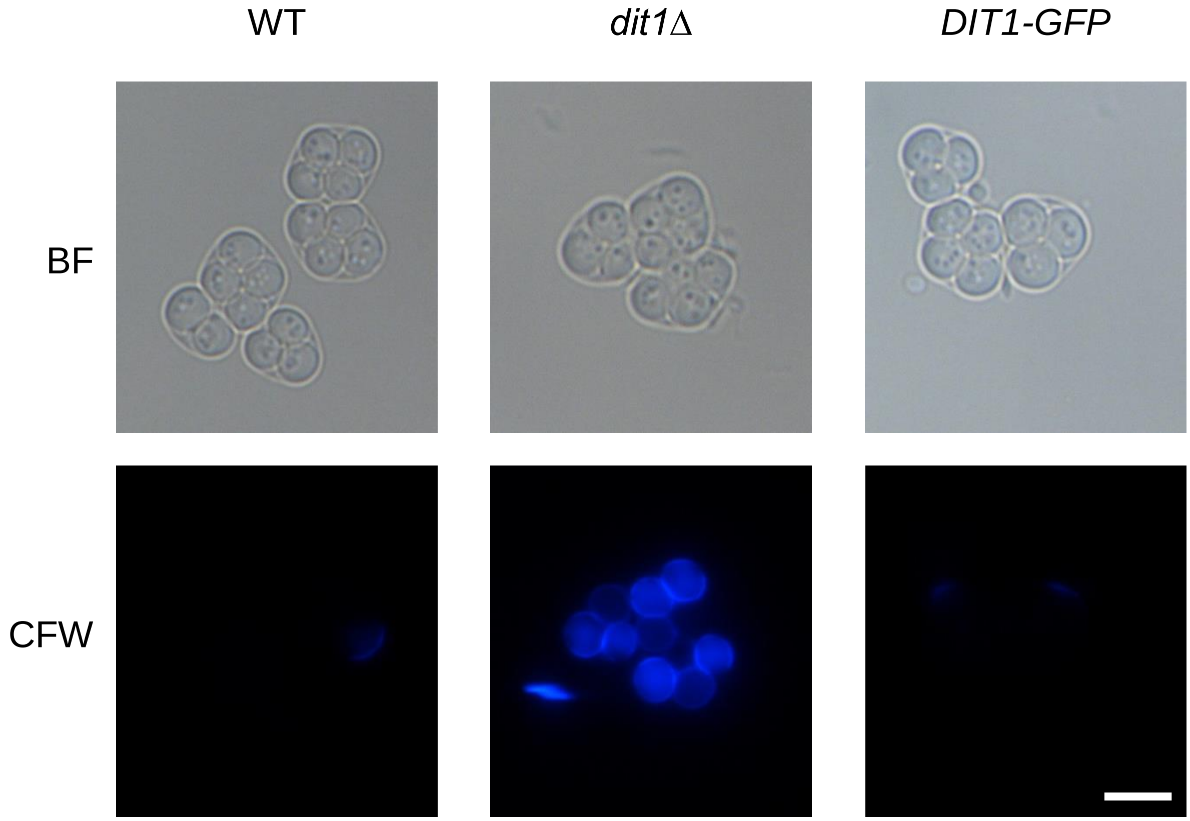

Figure S1. CFW staining of *DIT1-GFP* harboring spores . Wild-type (WT), *dit1Δ* or *DIT1-GFP* harboring spores were stained with CFW and observed by bright-field (BF) or florescence microscopy (CFW). Bar, 5 μm.

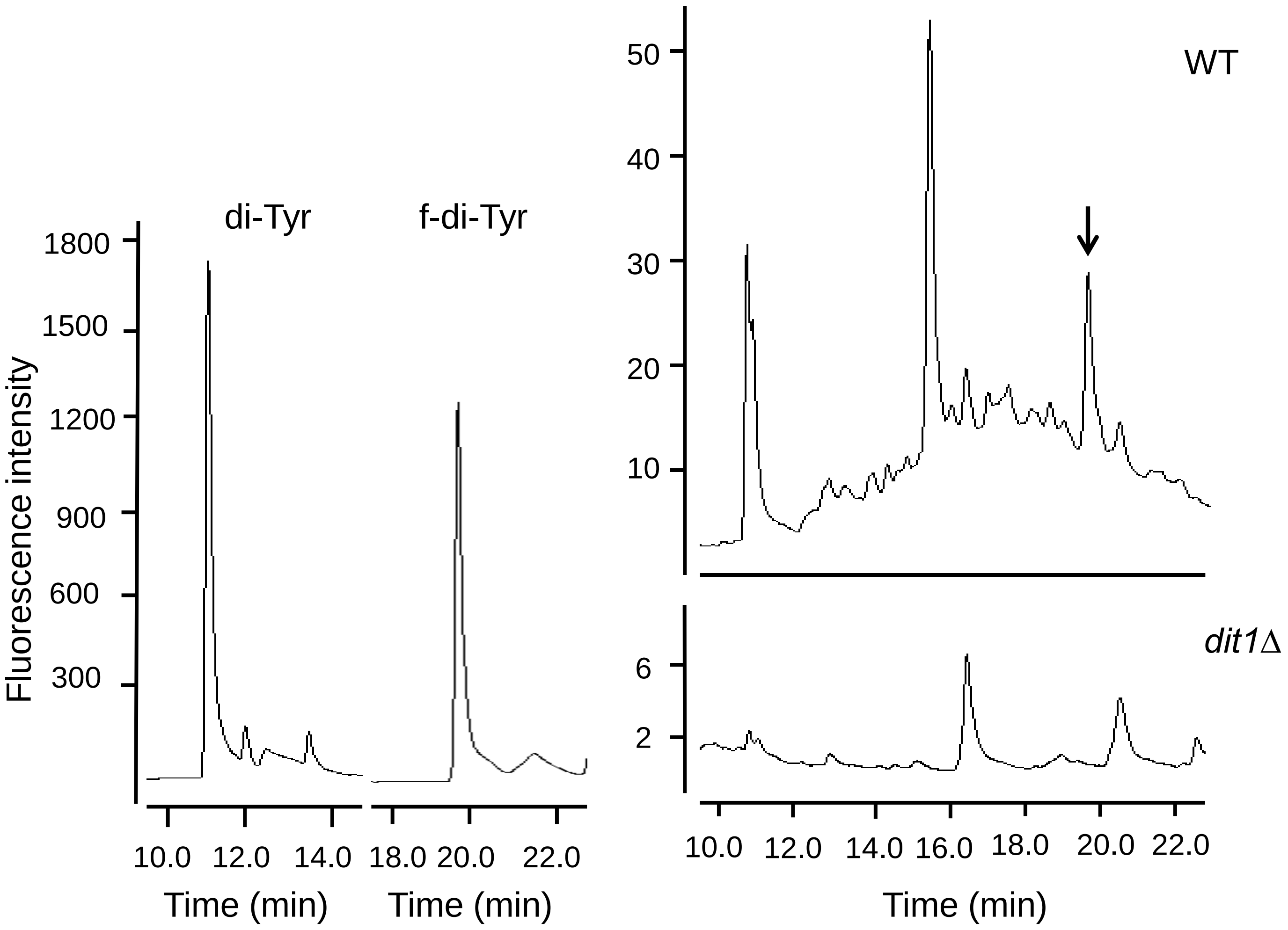

Figure S2. Trisodium citrate can release bisformyl dityrosine from the spore wall. Wild-type spores (WT) and *dit1Δ* spores were hydrolyzed with trisodium citrate and hydrosylates were analyzed with HPLC. The arrow indicates the formyl dityrosine peak after trisodium citrate treatment. Di-tyrosine (di-Tyr) and formyl di-tyrosine (f-di-Tyr) are shown as control.

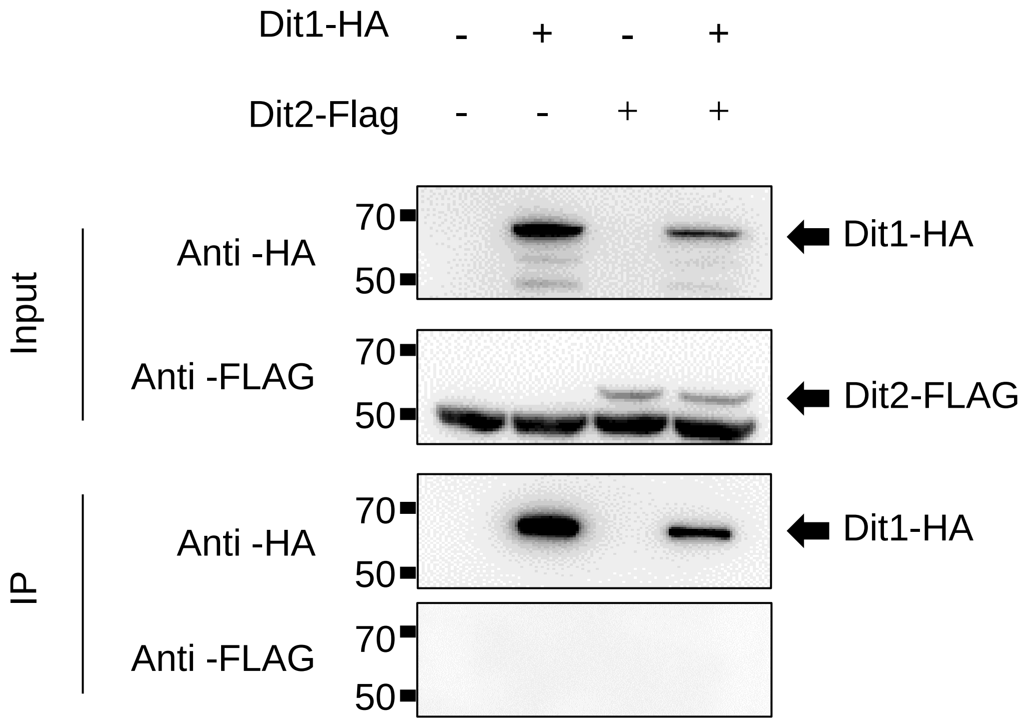

Figure S3. Co-immunoprecipitation of Dit1 and Dit2. Lysates from wild-type vegetative cells harboring pRS424GAL1-DIT1-HA (Dit1-HA) and/or pRS426GAL1-DIT2-FlAG (Dit2-FLAG) were subjected to co-immunoprecipitation analysis. The HA fusion proteins were precipitated with anti-HA-agarose, and the immunoprecipitates were subjected to western blotting analysis using anti-HA antibodies and anti-Flag antibodies (IP). 10% of cell lysates were subjected to western blotting analysis to show proteins inputs (Input).
